# Women’s Health in/and Work: Menopause as an Intersectional Experience

**DOI:** 10.3390/ijerph182010793

**Published:** 2021-10-14

**Authors:** Kathleen Riach, Gavin Jack

**Affiliations:** 1 Adam Smith Business School, University of Glasgow, Glasgow G12 8QQ, UK; 2 Monash Centre for Health Research and Implementation (MCHRI), School of Public Health and Preventive Medicine, Monash University, Melbourne 3800, Australia; 3 Monash Business School, Monash University, Melbourne 3145, Australia; gavin.jack@monash.edu

**Keywords:** age discrimination, intersectionality, menopause, occupational health

## Abstract

This paper employs an intersectional lens to explore menopausal experiences of women working in the higher education and healthcare sectors in Australia. Open-text responses from surveys across three universities and three healthcare settings were subject to a multistage qualitative data analysis. The findings explore three aspects of menopause experience that required women to contend with a constellation of aged, gendered and ableist dynamics and normative parameters of labor market participation. Reflecting on the findings, the paper articulates the challenges of menopause as issues of workplace inequality that are rendered visible through an intersectional lens. The paper holds a range of implications for how to best support women going through menopause at work. It emphasizes the need for approaches to tackle embedded and more complex modes of inequality that impact working women’s menopause, and ensure that workforce policy both protects and supports menopausal women experiencing intersectional disadvantage.

## 1. Introduction

In 2020, the Australian Government launched their Women’s Health Strategy 2020–2030, followed in 2021 by the Scottish and UK Governments’ Women’s Health Plans. While on opposite sides of the world, these policy positions and intentions signal increasing global awareness that economic participation is central to both the continued well-being of women and broader ambitions surrounding gender equality as articulated by the United Nations (UN) (Sustainable Development Goal 5, #gender equality) and World Health Organization (WHO). At the same time, evidence repeatedly highlights how work and workplaces can result in or exacerbate poor health for women across the life course, in part linked to gender-biased workplace cultures and the continuance of gender and age-based stereotypes [1].

As result of population ageing and a concomitant rise in workforce participation amongst women aged 45–64 years in OECD countries [2,3], large and increasing numbers of women are experiencing menopausal transition while in paid employment. This is because menopause (the final menstrual period) typically occurs in the age range of 45–55 years for most women, and on average, at 51 years [4] in Western societies. Menopause is often considered to have three stages—peri-menopause, leading up to menopause, and then post-menopause [5]. It is a process marked by ongoing changes in women’s menstrual cycles, and/or the onset of diverse menopausal symptoms (e.g., hot flushes, night sweat, sleep disturbance and problems with concentration). Studies show that women experience a range of vasomotor, psychological, physical and urogenital symptoms in response to different extents (e.g., more or less frequent, or more or less problematic/bothersome) [6], and that symptoms such as hot flushes and night sweats may last, on average, 7–10 years [7].

Contextual factors in the sociocultural environment shape and moderate symptom reporting. Key amongst these factors, as growing evidence shows [8], is the influence of the physical and psychosocial nature of women’s work and workplaces on menopausal experience. Limited qualitative work to date has investigated the nature of this influence as experienced through the prism of age- and gender-based inequality. The aim of this article is, therefore, to advance understanding of women’s menopausal transition at work through the lens of intersectionality. Complementing recent studies that have sought to depart from biomedical approaches to the menopause at work through feminist [9] or sociomaterial [10] approaches, this study focuses specifically on the ways in which menopause is experienced through multiple axes of power that presume a normative embodiment; that is to say, built around a series of age-related, gendered and ableist, and work status (such as full-time) ideals. Focusing on healthcare and higher education workers, this paper is relevant for the study of menopause at work given that these sectors have female majority workforces but also because they are institutions where women in general turn for support, research and information surrounding menopause. As such, it frames women’s health at work (that is, the experience of their health as employees within particular organizational contexts) as intimately connected with women’s health and work (the public messages and services that create new knowledge surrounding menopause and work and seek to encourage women’s participation in the labor market through supporting their health).

### 1.1. Women’s Health in/and Work through an Intersectional Lens

Intersectionality is a theoretical lens, most commonly associated with Crenshaw’s [11] work on race and women, that also includes a consideration of class and sexuality in relation to women’s subjectivities and lived experiences of oppression. Rather than considering these as identifying features, Crenshaw terms them ‘axes of power’ [12] (p. 787) to emphasize how their organization and subsequent marginalization is a socially situated rather than individual phenomenon. In rendering visible the manifold consequences that ensue, Crenshaw [13] highlights the way that inequalities are not simply additive but cumulative and rebound across private lives and public institutions. In other words, the experience at various nexuses of gender, race, class inter alia is more than the sum of the parts of, for example, sexism and racism due to structurally embedded mechanisms of inequality. Work is a significant site where this manifests, as shown in the focus on employment law in Crenshaw’s [11] early writings.

Recent accounts of reproductive health have highlighted both the manifestation and consequences of intersectional disadvantage. Access to healthcare and subsequent treatment surrounding pregnancy has long been established as subject to gendered racist totems [14]. Elsewhere, emphasis on the intersection of disability and reproductive health created enduring conceptions of ‘normalcy’ that systematically denigrate both men and women, although this manifests in different ways across genders [15]. Studies of menopause also articulated differing experiences amongst women in consonance with gender and/or age, and/or sexuality, amongst other hierarchies [16]. More recently, calls to understand and respond to the effects of COVID-19 emphasized the need for an intersectional lens surrounding the health life course of women more generally [17].

At the same time, there is an established body of literature exploring how women at work experience intersectional disadvantage in composite with race [18,19,20], sexuality, disability [21] and class [22]. In particular, studies of embodiment at work have highlighted how workplace inequality manifests through particular bodies being indexed as more or less valuable or capable. Of importance here is work exploring ‘gendered ageism’ whereby feminized ascriptions create unattainable and fantastical expectations surrounding ‘agelessness’ [23] or problematic stereotypes of ‘cranky old women’ [24].

Such studies highlight the fertility of an intersectional lens academically. Yet unlike other theories of inequality that are unfortunately often consigned to academic journals, Crenshaw’s term is now a mainstay of how policy, practice and general public discourse feed into experiences of bias or injustice.

### 1.2. Women’s Health in/and Work in Healthcare and Higher Education Sectors

Women’s health across the life course is an important topic of concern for both healthcare and education sectors. In Australia, women comprise 57% of the higher education sector [25] and 78% of the health and social care sector [26]. This means that women’s health as a life course phenomenon should be central to how the workforce is managed, supported and structured more broadly. In particular, menopause is a growing area of attention for managers and support functions within the healthcare and education sectors, as both workforces comprise a high number of older women. For example, the typical age range for menopause in Western settings is between 45 and 55 years, and an average of 51.2 years [19], while 46% of nurses in Australia are 45 years or over [27]. Previous evidence within school educational settings, where the OECD average age of a teacher is 44 [28], showed that women experience negative perceptions or have had their menopause problematized in a workplace context [29].

In beginning to explore the intersectional nature of menopause, it is important to note that experiences are likely to be influenced by broader historical dynamics within these sectors. For example, COVID-19 has provided a timely account of how institutional practices within healthcare rest upon historically gendered intersections. Here, a predominantly low-paid female healthcare workforce (in the form of nurses, healthcare assistance and domiciliary staff) has been at the frontline of not only taking care of the population, but also called upon to be surrogate and proxy members of the family, birthing partners and companions in death, due to restrictions on hospital visitation. Such aspects speak to the historicized position of women in low socioeconomic positions assuming a burden of care. They also highlight the duality of how historically situated modes of intersectional inequality manifest in contemporary practices, or, as Crenshaw [30] (p. 15) states: ‘the integration between what is structured—the historical and enduring institutions that constitute and naturalize social power—and what is dynamic—the ways that power is continuously reproduced and contested in real time’. This dimension is particularly important in relation to menopause given that health, as both an institution and a mode of cultural and social practice, is heavily instituted by the ways in which individuals work and seek to make a living.

Yet the value of considering women’s health in/and work as an intersectional phenomenon is that it highlights the complex and ambidextrous ways that work and health manifest in multiple but overlapping spaces. In particular, the in/and work elements mean recognizing a series of tensions that exist between advocating for and supporting the health and well-being of women in the general population, while at the same time ensuring that the workforce charged with this duty is also supported.

### 1.3. Menopause in/and Work

Early studies surrounding the menopause at work considered menopause as a variable that could either negatively impact work, or work as an explanatory variable for how women experienced menopause [8]. However, the past 10 years has provided a wealth of new evidence that creates a more nuanced account of menopausal experience in/and work. More broadly, it has been situated as part of organizing systems that disadvantage women across the life course, as captured in Grandey et al.’s [31] account of the ‘three m’s’ in women’s lived experience of work (menstruation, maternity and menopause).

Most recently, Atkinson et al. [32] emphasized the need for what they term an ‘intersectional political economy’ approach to understanding menopause at work. This extends accounts of the lived experience of menopause at work as a gendered phenomenon within a particular set of power relations surrounding ethnicity [33], and situates it within broader circuits of power, resources and control at a meso and macro level. Such a politically sensitive account is particularly welcome in highlighting the uneven geographies of menopausal experience and how they coalesce with other dynamics surrounding labor.

Such a review provides a valuable backdrop for highlighting how the menopause at work can sit at the intersection of gendered, aged, classed and racialized dynamics. At the same time, there is less specific focus on how women themselves experience menopause as an intersectional phenomenon as subject to different organizing structures that are constituted by multiple and intertwined power relations. In other words, if menopause is an intersectional phenomenon, what are the consequences of experiencing it as such within a workplace context?

## 2. Method and Materials

### 2.1. Research Design

The analysis is based on 1929 open-text responses that followed a survey about health, well-being, menopause and work by women working in three organizations in the healthcare sector and three higher education institutions. In highlighting key themes from the data that explore the intersection of gender, age, health and work, the article considers women’s health in the workplace in a way that takes into account interpersonal, cultural, environmental and professional considerations.

Five of the six workplace settings were based in metropolitan areas of Australia and one was in regional Australia. All organizations employed at least 1000 people. Access for the research was secured through engagement with senior management and Human Resource leaders in each of the organizations, although, as per ethical guidelines, they were unaware of which employees had participated in the research.

The interest in the open-text responses was to give space for open reflection and critique beyond the deductive approach used in the survey instrument, while providing a broader landscape of ideas that could help round out the more in-depth (but small numbers) of interviews undertaken. Complementing recent interview-based studies of menopause at work that have focused on an interpretivist approach [9,10], this research adopted an phenomenological approach, which has gained popularity in exploring women’s health in other spheres [34,35]. Here, ‘If one wants to study the world as lived through, one has to start with a ‘direct description of our experience as it is’ [36] (p. vii)’. In other words, an individual’s reflections on their lived experience of a given phenomenon provides the analytical inroads into how the world is constituted [37]. In particular, the research project sought to explore how women themselves framed and narrated their experience of menopause at work away from the researchers’ own preconceptions or expectations. Gaining an insight into the practical negotiations and everyday accounts of women was important in exploring the intersectionality of menopause given that Crenshaw emphasizes how ‘practice necessarily informs theory, and how theory ideally should inform best practices and community organizing’ [12] (p. 786). In other words, further theorizing surrounding menopause at work can be informed by what women themselves do and how they experience menopausal transition while working.

### 2.2. Data Collection

For the higher education cohort, women from administrative, academic and executive roles were surveyed. A total of 971 open-text responses to two questions were completed from the total survey number of 839 respondents. Data from these responses were notable in terms of the level with which people engaged in the questions, with a total word count of 21,928 and addressing a wide variety of experience of both themselves and their immediate colleagues. For the healthcare data, 958 open-text responses to three questions were gathered from a total survey number of 1092. These responses were shorter, in general, per response, but still constituted a word count of 24,323 words.

An important decision during data collection was how to set parameters surrounding menopausal transition. There are medical scales that are used within healthcare settings to categorize menopausal transition (e.g., [38]), while others use chronological age as a proxy for how ‘close’ women are to the average menopausal age. For the analysis of the open text box data in this paper, we used the classification on the STRAW +10 Staging system [5,38]. Women were asked a series of questions about their menstrual status. From these responses, women were categorized into three menstrual categories (pre-menopausal, peri-menopausal and post-menopausal). This system categorizes women into menstrual stages depending on when they had their last period and the variability of their menstrual cycle. At the same time, there was analytical consideration of how women self-identified their menopausal status. This was, in itself, insightful as many respondents were not clear about their own menopausal status. For example, there were a number of statements such as ‘not even sure if I’m there yet’ (HE121), ‘I don’t think I am menopausal (or am I in denial?)’ (HE750) and ‘I am not experiencing any signs of menopause—at least none that I am aware of’ (HE396). Others discussed symptoms but did not place themselves into one of these classifications, with comments such as, ‘Is fatigue a perimenopausal symptom?’ (HE247) or ‘Not sure, I’ve only recently (2 weeks) noticed the hot flushes and so am only just beginning to explore options’ (HC990). This ambiguity spoke to a broader discussion surrounding what menopausal transition might involve in terms of symptomology and health experience, as summed up by one respondent below:

*So interesting to read your check list about menopause. Shit! Are they all symptoms? Really? The palpitations? The dizzy stuff? The anxiety? I am going to make an appointment to go to Jean Hales (menopause clinic). You know, I always kind of thought menopause was just about the sweats. They are uncomfortable—but not the end of the world. This other stuff—tiredness and the anxiety and the anxiety like symptoms are shit* (HE509).

### 2.3. Data Analysis

The data were qualitatively analyzed using a three-stage process. The first stage involved the coding of each corpus (Healthcare—hereafter referred to as HC in findings followed by respondent number; and Higher Education, hereafter referred to in the findings as HE followed by the respondent number separately), with first-order coding techniques [39] used to inductively identify specific themes. The coding books from each corpus were brought together and amalgamated, with similar codes consolidated. The second stage involved exploring the patterns and relationships between codes, although we specifically chose to avoid exploring relationships between the initial coding of social categories so as to avoid an additive (rather than intersectional) approach [40]. Instead, we identified which other codes overlapped with each other that may have suggested a causal relationship between particular ideas. In some instances where the free-text comments were longer, it was possible to do this within particular extracts. For others, it involved looking at different codes that operated in a constellation around a single code within different responses. For example, reference to temperatures was analytically connected to codes around lack of control, while for others, this was connected to exacerbating symptoms, and for some, codes around gender-hostile workplaces. Doing so helped to create what the research team termed ‘coded trinities’ whereby different power dynamics were identified. This was particularly useful as it allowed us to begin seeing connections to the ways in which inequalities and power manifested within the context of work environments, conditions and cultures.

Once the analytical process had established these trinities, the third stage involved returning to the original data and looking at how these institutional spaces, cultures and conditions (as reflected in the coded trinities) could be used to further understand the descriptions that women had given of their menopausal experience at work. At this point, we returned to the original data to confirm whether the analytical trinities were reflective of the responses from both peri-menopausal and post-menopausal women. During this third stage, careful attention was paid to engage in an open reflexive inquiry to explore the research team’s situated positionality and possible biases. Part of this involved paying attention to a number of elements that Vardeman-Winter et al.’s [40] work on HIV in indigenous rural communities in Canada highlighted. These included interrogating how comparisons between people were being made, attention to a pull or desire towards universality or the essentialization of experience, and a constant attention to context.

## 3. Findings

The analysis identified three areas where women experienced menopause as an intersectional experience, as outlined in Table 1 and further developed in the following sections.

### 3.1. Menopause as a Slippery Inequality

To begin articulating menopause at work is to consider it as a simultaneous health, social and cultural experience. Yet the majority of the accounts given rarely identified only one aspect as an issue without relating it or contextualizing it within the broader dynamics of work or their personal lives. For example, one woman in a university stated: ‘It is difficult for me to judge whether peri-menopause is affecting my capacity to deal with a range of issues (depression stress etc.) because of other things going on in my life at the same time’ (HE82). This implies that it is difficult to attribute what was a ‘menopausal’ experience at work from the broader experience of workplace interactions and unequal practices.

An example of this relates to the normative expectations of what is sanctioned as ‘correct behavior’. Many accounts suggested that women’s behavior in the workplace was viewed as menopausal or problematic as a result of gendered ageist norms. For example, one woman suggested there ‘needed to be an acceptance that women can be assertive or argue without judging them differently to men’ (HC431). In this sense, many respondents were concerned about disclosing their menopausal experience as menopausal at work for fear it would be used against them. One suggested that their organization would ‘use menopause as an opportunity to criticize or demean my functional part in this unit or constantly remind me of adverse events and use them to pursue other agendas’ (HC128). In other words, ‘menopause at work’ as a phenomenon carried the danger for women of intensified modes of discrimination, by virtue of being a middle-aged woman at work. This resulted in fear to disclose any health-related experiences or ‘weaknesses’, with one healthcare provider stating that ‘I would feel ashamed and frightened to expose the extent of my health problems as it may impact on how I’m viewed’ (HC947).

As suggested above, the slipperiness of attributing experience to menopause also occurred amidst a constellation of other longer-term health experiences. For those experiencing menstrual health conditions, menopause was often framed as ‘just’ another chapter in self-managing their health as a working woman. For example, one respondent stated that: ‘Dysmenorrhea (period pain) associated with endometriosis has been a constant problem impacting on my work experiences throughout my entire career’ (HE366). Responses suggested that these health episodes were rarely taken seriously, with one respondent arguing that management needs to be ‘encouraged to think of menopause and other problems such as dysmenorrhea, endometriosis etc. are real physical problems and cannot be treated with a “mind over matter” attitude’ (HC43). Trying to extrapolate menopausal experience at work from other conditions was both challenging and, for many, unhelpful, and others discussed how their menopause was experienced in combination with managing other pre-existing conditions:

While other health conditions and experiences that occurred at the same time as menopausal transition were unique for every woman, there were two patterns that repeatedly emerged across the corpus that highlighted the challenge of menopause being viewed in isolation. The first experience related to long-term conditions that had always been affected by work factors but were further exacerbated during menopause. Here, women disclosed that such experiences were often combined with feelings of failure or isolation. Many discussed letting colleagues down or not putting themselves forward for jobs due to not knowing how perimenopause or other health conditions would affect them. For example, one woman experiencing health issues that had ‘become more prevalent since the onset of menopause’ stated ‘I have to change floors to get a cup of coffee… there is a lift but I am aware of the looks if I use it. I simply cannot join the walking group or take part in the 10,000 steps because I am a liability to a team score and I don’t meet the targets’ (HE311). This was particularly the case for those who had gone through early or induced menopause. Such experiences were framed as happening ‘out of step’ and not being understood by colleagues. They also portended financial concerns due to menopause happening at an earlier time in their career than expected, as well as feelings of not fulfilling age-expected behaviors:

*Lymphoma in 2011 has caused me to continue to be tired post treatment, has brought on early menopause, has increased emotional problems within my family, sometimes impacts on my emotional status (worry about future) has impacted on us financially. Due to my past treatment for cancer I have reduced to part time and this impacts on my ability to complete work tasks. On a positive note it has encouraged me to have more work life balance, to spend more time with family and to make my time at work as efficient as possible. I have focused on making work and processes transparent and as simple as possible* (HE98).

*I am 47 and I went through menopause in my 20s but was not diagnosed until I was nearly 30. I was encouraged to take HRT which created some menopause-like symptoms and other side effects. Any menopausal symptoms I had, I had a long time ago and I’m glad they are gone. It was difficult coping with it at a younger age when people don’t expect you to be unwell* (HE640).

It was somewhat surprising (although perhaps less so to those working in healthcare) that respondents working in healthcare, and sometimes in wards directly associated with their conditions, had reported less sympathy for their responses to treatment. This was often attributed to increasing pressures of work through budget cuts that made managers forget that employees were ‘fellow human beings’ (HC284). At other times, women discussed direct hostility towards being ill. For example, one woman (HC722) who had her ovaries removed, which had ‘effectively put my body into shock and early menopause’, was warned about taking sick leave and ‘when I informed her I was unable to walk properly due to the stiches and had to take longer off all she could say was “well you could always take the elevator if you came back to work”’ (HC722). Women in higher education also experienced negative perceptions, with one stating: ‘I find it difficult to get support or express the need for support at the workplace as gender related health issues such as this could be perceived as a weakness and could provide the basis for lack of promotion and ability to perform. I definitely feel older women are disadvantaged in the workplace’ (HE269).

The second pattern related to the experience of health conditions as either exacerbated by, or brought on by, menopause, and subsequently causing negative workplace experiences. This was often discussed in relation to depression, anxiety and other mental health conditions. For example, menopause or prescribed treatment had an impact on pre-existing conditions that, in turn, impacted their experience of work.

*I’ve experienced menstrual irregularities this year that have warranted medical attention, although technically I’m not considered menopausal, or close to it. The medical solution affected my depression, which affected my capacity to work, so yes, indirectly I think my lead up to menopause has impacted on my work performance, and I’m dreading the next few years of getting through this stage in my life. I’m lucky to have an understanding supervisor* (HE253).

For some, a desire to continue performing as expected in the workplace impacted their broader interactions with healthcare professionals. One woman described being prescribed anti-depressants for what she termed ‘mental fatigue’ although ‘I don’t really relate to classic symptoms of depression’, continuing to state ‘I am not sure if menopause or the medication affects my memory’ (HE380). Other women discussed their decision to ‘opt out’ of a continuing healthcare discussion with their GP due to fears about how it might impact their performance at work, and the experience of being pressured to be successful in work as counter to their experience of health:

*When I went through menopause I became depressed for the first time in my life and so I had time off work and did take anti-depressants for three months. I went off them as the doctor wanted me to double the dosage and I was worried that I would not be able to function so went off them and stopped seeing the doctor* (HE55).

*Very heavy periods are becoming problematic and I am worried that I won’t be able to find enough time in a heavy work load to plan a hysterectomy (elective). This really detracts from my quality of life and sometimes it gets me down. Also, my mother died at an early age with cancer and I am worried about my longevity—so I feel an urgency to get on with my career and to succeed* (HE794).

*I suffered extreme tiredness, palpitations and other symptoms that I put down to peri-menopause and too much work (I hadn’t taken a proper break for well over a year). I didn’t go to my GP because I was so busy. When I eventually did, for something quite different, she sent me off for a series of tests that discovered a very severe iron deficiency caused by heavy peri-menopausal bleeding. I was annoyed with myself for not having seen her earlier, but I think it was very much a case of ‘soldiering on’ at work and ignoring my own needs because of an unrelenting and high workload, and my feeling that I had something to prove at work (that is, that a fifty-two-year-old woman is as good as anybody else)* (HE809).

Others discussed feeling guilty about their perceived underperformance, which they worked late to make up for (HE161). In seeking to counterbalance work expectations with health implications, many women were faced with a difficult choice around how to negotiate a workplace and menopausal transition without negative consequences. In this sense, it was more the need to respond to or negotiate menopause under certain cultural and organizational conditions that led to a number of respondents emphasizing how they either had ‘no time with energy to myself’ (HC19) or ‘no longer feel like me’ (HE375).

### 3.2. Laboring through Menopause

For many women, their menopausal experience was part of a broader story around finding themselves in gendered and aged positions in the workplace that often involved significant levels of emotional labor or complex people management. Rarely did they feel that the effort or the skills required were recognized at an institutional level and the stress of this particular type of work could exacerbate anxiety and stress levels—symptoms associated with menopausal transition [6]. Many respondents framed this as ’hidden’ work that their older male colleagues were either not expected to do or would refuse to engage with. By comparison with older women, men were seen as being given support for promotion and other aspects that aid advancement such as ‘access to strategic information much more easily than women’ (HE398). However, if the women chose to challenge this, their voice was often dismissed or attributed to a problematic menopause:

*There is the very real issue of over work, and lack of gender awareness, particularly by the men in the workplace. This is not directly related to menopause, more a perception that women will pick up the pieces and do much of the “hidden” work, and men get kudos as Head of Department, Chairs, Associate Professors etc. These systemic problems are gendered, which contributes to stress in the workplace* (HE369).

A number of accounts also discussed how menopause coincided with a broader gendered ageism embedded into work cultures and a number of women framed their account in terms of growing older, and ‘feeling invisible’ (HE31) and ‘too old now to be of interest to people generally’ (HE525):

*I feel like I have been consigned to the scrap heap in most facets of my life, i.e., passed my “use-by date”. I feel I have reached that “invisible woman” stage of my life and that if I assert myself then I am perceived as “an old hag” or a “difficult woman”* (HE375).

For one respondent, this also played out due to hierarchies of ethnic privilege as well, where ethnic minority women reported being overlooked in favor of both white women and ethnic minority men for career development opportunities:

*My line manager has not spoken to me in over a year; I see male colleagues of same origin as male supervisor get training which improves promotion and I have more experience and am left “to rot”: other male colleagues just ignore me even when I’m placed in an overseeing role but the top manager, which in my opinion is sex related too* (HC949).

Many of the respondents mentioned wanting to go part-time as one way of taking control of their health, lifestyle or work–life balance. For example, some had ‘returned to nursing on a casual basis so I can control the number of shifts I work’ (HC139). However, some felt the stretch on the higher education or healthcare sectors meant they did not want to put colleagues under additional pressure since it was unlikely that their ‘fraction’ would be replaced. For example, one suggests ‘I would quite like to work four days a week but due to staff numbers would not ask!!’ (HC459). For others, the decision was more driven by personal circumstances and financial demands, especially for those on casual contracts or of lower grades. For example, one respondent discussed anxiety from menopause as intimately connected to ‘my lack of financial security, due to inconsistent nature of casual contracting, plus worry about two teenagers’ (HE22) or ‘financial concerns related to my current contract’ (HC1137). A number of other women also discussed needing to support partners:

*I feel a lot more vague and forgetful e.g., muddling up the times of appointments of making mistakes with practical things like travel arrangements. I also find the hot flushes exhausting and sometimes embarrassing. To compound matters, I have carer’s duties for a frail parent, and I am in the midst of a lot of extremely distressing family conflict. If I weren’t the sole income earner, with a dependent spouse with some mental health and associated substance abuse problems, I’d definitely go part-time* (HE568).

Some felt that they were ‘trapped’ into staying full-time due to the risk of losing benefits, with one suggesting: ‘I feel I could continue working for another 2–3 years if I could reduce my hours to part-time’ (HC588). This appeared to have been accurate according to the accounts by those who were not full-time. For example, respondents suggested that they were not given development opportunities for training, even though there was a recognized need to support older staff in terms of new initiatives and practices (HC898), while others spoke about how they were assigned more difficult roles due to being part time. Others disclosed that their part-time status created a different attitude to how their health was treated:

*Being a part-timer, I am expected to deal with any appointments in my own times. I was told that being full time has certain benefits, e.g., rostered days off* (HC14).

### 3.3. Decentering the Self: Menopausal Inequality through the Ethos of ‘Service’

The final set of findings highlights how menopause as an intersectional experience is organized around an ethos of ‘providing a service’. On one level, menopause was, for the respondents, directly impacted by the working patterns required within their sectors, particularly in healthcare work. This often revolved around shift patterns or spaces available for working:

*Because of my age, menopausal symptoms and poor sleep I find it extremely difficult to do night shift and get home safely and sleep well. I have already ran off the road and crashed my car on the way home from night shift but no one cares. I still have to do my share of night shifts* (HC22).

*Personally I cope well with menopause. Have witnessed increased anxiety in others. More frequent breaks, ability to sit uninterrupted to do the increasing paperwork required. Most nursing environments have totally inadequate desk space, even tables to rest paperwork whilst writing. Also need an opportunity to put feet up/elevate when on break without feeling rude!* (HC348).

*Night duty. This contributes enormously to my sleeping difficulties at my age. When young I had no difficulty sleeping with night duty* (HC127).

Such accounts suggest that certain workplace conditions created inhospitable environments for menopausal women, such as room layout or temperature where, in one instance, ‘air conditioning (is) set for normative bodies’ (HE190), and in another, open plan or connected offices made it impossible to control the temperature: ‘(We need) better temperature control—for instance a change in the thermostat in my office sends the office next door to arctic conditions ‘(HC513). Others discussed problems with uniforms, including scrubs and ‘the blue gowns that we have to wear because of the VRE Van A & B strain. The gowns have no insulation and make us all very hot, especially if you have to wear it longer than 5 min’ (HC739).

Across both healthcare and higher education, these structural and environmental aspects had consequences in terms of how the women negotiated practical symptoms against cultural workplace dynamics. However, this negotiation was not only about physical discomfort but forced the women to make a decision about being uncomfortable in saying something, for fear of ‘outing’ their menopause or causing discomfort to other members of the team. Some discussed how managerial and supervisory structures made this easier and emphasized how sympathetic line managers and supervisors were vital for helping to successfully negotiate menopausal symptoms while working. However, even in these cases, it could be that broader workplace structures did not provide a way of best supporting them. For example, respondents discussed how their anxiety had been exacerbated not only due to menopause but because of the way their workplaces were organized in terms of a lack of accountability or support:

*The combination of no structured leadership and supervision, combined with being a senior person working at part time days, family responsibilities and menopause has been hard. I frequently feel like I’m doing everything half- well* (HE358).

In both the healthcare and education settings, respondents noted that their menopausal experience highlighted the disconnect between the increased notion of care for service users (either patients or students) and the treatment of employees, and the inability to ‘act out the values instead of preaching about them’ (HC790). For many, it was the increased emphasis on consumer-orientated discourses about ‘service users’ and ‘customer expectations’ that resulted in the toll on their bodies:

*My organization works hard to achieve excellent customer service, but I hope that employees’ working conditions will also be given equal importance* (HC15).

Given that, in both sectors, front line staff were majority female, the increased burden on service expectations and student contracts fell to many of the respondents within their organizations. However, this additional work involved in satisfying new patient or student charters was often absorbed into existing workloads. Against this backdrop where workers were encouraged, through structure or ethos, to displace themselves for the sake of the patients or students, a number of women emphasized that their menopause was something to be hidden alongside broader identifying features of age:

*My colleagues do not know how old I am, I keep this very secret, as there is a tendency for people to consider the ‘young’ ones as being more capable with technology. I have had the experience of people trying to trick me into revealing my age. Terms have been used (even from colleagues younger than me in their 50s), ‘young pups will be able to do this’ get those ‘young librarians involved’ (particularly if the task has to do with new technologies of which I am quite comfortable with). I find this very insulting (which only encourages me further to hide my age), these comments come from peers and management. I have heard this on a number of occasions and think that if I anyone knew I was over 60, immediately I would be considered almost retired and people’s attitudes would change* (HE254).

For many, the physical space in which they worked could make the choice to ‘hide’ their menopause more difficult or easier. For example, many discussed their ability to work at home as helping their menopausal experience at work. One respondent (HC699) suggested a number of ways to ‘hide my worst symptoms from colleagues’ such as ‘flexible working arrangements e.g., work from home, temperature control for the immediate working environment’. Another woman disclosed that: ‘For me, it’s a private issue but I’m fortunate that I have my own office I can retreat to. I’m not sure many women who like to be “identified as menopausal”’ (HC392). For others, the nature of their work tasks made it difficult not to disclose, even if they wished to, making the irregularity of peri-menopausal symptoms such as ‘flooding’ (heavy bleeding) difficult to ‘hide’:

*My periods are variable—can occur anytime—been in embarrassing situations a number of times at work—very difficult to deal with as once visible blood leak onto skirt during a lecture* (HE837).

Others suggested that this intensification of work was also insensitive to broader challenges surrounding how unprepared workplaces were for employees growing older at work. In particular, many respondents suggested that career development or flexibility in how to work that was mindful of the effects their work had exerted on their bodies over 20 or more years was lacking. This was connected to a broader awareness of their respective sectors being ‘age-blind’ in their structures and practices:

*The workforce is ageing and yet no measures seem to be in place to help 40+ nurses, railroaded and alienated by younger staff in management positions such as NUMS (Nursing Unit Managing Supervisors)* (HC623).

*Workplaces generally and our community overall appears to be in a period of transition and/or confusion regarding the rhetoric surrounding work/life balance and the day to day realities and challenges of the workplace to facilitate such a balance. The issues may vary depending on the age of the worker e.g., starting out in the workforce, young families, mid-life, approaching retirement etc. and the plans put in place by the individual for post work life are subject to major external change e.g., changes to the age of retirement, superannuation rules and regulations. But to date our workplace structures are struggling to adapt to the life stages in the extended working life of the individual (both male and female)* (HC730).

This awareness was also required to ensure that workplaces were more age-friendly in general (HC518), with a number of women suggesting that symptoms associated with menopause were also common amongst men. For example, HE775 suggested that ‘I feel a loss of cognitive ability, but I think it is age-related rather than menopause as my husband experiences the same things’, while others spoke about symptoms of menopause being the same as symptoms of being overworked and too busy. This was situated not simply as an organizational problem but as reflecting part of a broader landscape that perpetuates age biases where ‘*** (name of organization) is the same as the rest of society in displaying undercurrents of ageism’ (HC844).

At the same time, women were keen to emphasize that against this backdrop, support should be reflective of the experience of menopause itself in terms of recognizing that, for most, menopause is a transition and that any changes to their workplace behavior are not an indication of longer-term performance. As one respondent said: ‘I am conscious of being a little more scatty and forgetful, which I gather will come right and I am really looking forward to that’ (HE788). Similarly, other women emphasized that organizations framing menopause as an episode or transition could help to highlight that an employee’s valued contribution over the longer term was recognized, and that menopause in no way precluded them from planning the next 15–20 years of their working lives. As HC102 reflected:

*Recognize and acknowledge that it will not always be this way for your employees. That is, just because someone is having some difficulty because of menopause—it would be affirming to know that the employer realizes that this will not always be like this and it will change as symptoms subside (e.g., confusion)—that this is usually only temporary.*

## 4. Discussion

This paper advances the understanding of menopause at work as an intersectional phenomenon. In one sense, this complements previous studies of a gendered life course, echoing ideas that age-related discourses are gender-specific and intimately attached to their embodiment, whether that be in earlier [31] or later [16] working life. However, more significantly, an intersectional lens helps to take away a focus on women’s menopausal experience as an individualized ‘problem’. Instead, it draws attention to dynamics of power that circulate in organizations and inform structures, environments, policies and practices that marginalize and oppress particular kinds of bodies and ways of being in the workplace. While symptoms of menopause may need to be negotiated within a workplace context, far more challenging are the inhospitable and embedded norms and cultures that women face day-to-day.

### 4.1. Intersectional Dynamics Impacting Menopause

The findings demonstrated the value of an intersectional approach in that it provides opportunities to consider women’s health at work more broadly as an ongoing negotiation of organizational priorities and power. The consequences and effects of menopause were difficult to isolate from other dynamics surrounding sexism, ageism or ableism. In some ways, this was important as these elements provided crucial ways for women to articulate their embodied experiences of the workplace. Until recently, menopause was stigmatized to the extent that there was little social and cultural conversation about its experience beyond close relationships, which were often considered ‘women’s business’ [41]. In this sense, the language of sexism, ageism or ableism provided respondents with a scaffold and language through which to make meaning of and articulate one’s menopause at work as intersectional, even though this may have only partially captured this experience.

While this narration is important for a politics of recognition, it provides an insight into the challenges in trying to redress menopausal inequality more broadly in employment policy and law. This is particularly important given that in a majority of Global North countries, claimants or complainants are required to ‘opt’ for only a primary mode of discrimination (such as sex, age or disability). As Crenshaw’s critical account of policy discourse surrounding race suggests, the influence of what becomes the ‘common, if not dominant, frame for addressing the disparities’ [11] (p. 154) can also silence a plurality of intersectional effects. In other words, framing menopausal inequality as only arising from gender, age or disability means the full force of intersectional disadvantage that the respondents disclosed in this article is difficult to capture within formal procedures such as employment tribunal settings and equality legislation.

The findings highlight the value of an intersectional approach in situating menopausal inequality not as an individual condition, but rather, problematizing it through the enduring imprint of gendered aged expectations within broader historical and sector-based foundations that subsequently organize working women’s menopausal experience. These ‘overlapping structures of subordination’ [12] (p. 797) manifest through women as a ‘flexible female’ [33] who are positioned as ‘putting up’ with particular conditions that decenter their own health, wellbeing or lifestyle needs. These sector-specific effects highlight how menopausal inequality is experienced through an intersectional ‘prism attuned to localized patterns of thought and action’ [13] (p. 307). However, the findings build on recent studies that employ an intersectional lens to explore menopause [32] through highlighting how these effects manifest in the simultaneity of intimate, lived, personal consequences and broader economic consequences for themselves and the workforce more broadly.

At the same time, it is important to acknowledge that menopause itself is experienced differentially by women by virtue of a range of other indexes around where, how and under what conditions they work. The respondents were clear that these elements were often the difference between both being able to choose to disclose their menopause in work, and how they experienced their symptoms as manageable or otherwise. However, it is noted that these elements of workplace flexibility, freedom and autonomy are usually consigned to professional occupations where middle class white women are disproportionately represented vis-a-vis minority women or those from lower socioeconomic backgrounds [42]. It would be valuable for future studies to further explore other sectoral dynamics to ensure that research evidence surrounding the embodied experience of workplace menopause being that of a professional white woman does not itself inadvertently sideline the experience of those with less power or voice, either within the labor market or more generally.

### 4.2. Implications for Practice

The accounts from respondents suggested that the deeply embedded modes of gendered ageism or gendered ableism within organizations are central to why many women wanted to keep their menopause as a private or unspoken experience. Throughout the findings, it was clear the multiple layers of inequality that constituted menopausal experiences at work resulted in a certain cynicism towards some of the rhetoric surrounding menopause support at work. Within this context, the fear of being ‘outed’ resulted from this rhetoric resting upon (rather than challenging or dismantling) inhospitable working environments and practices. It is important for organizations to embed support for menopause in unobtrusive ways that make public disclosure a choice rather than a condition to access support. For example, this could be ensuring that structures are in place where women only have to tell a small number of people in order to discuss menopausal support. Further responses include reflecting on whether a specific menopause at work policy is the best way forward, rather than integrating menopause as an included criterion across a number of other policies. Many supportive practices can be made available to the workforce as a whole, meaning that women do not have to disclose or ‘confess’ to menopause in order to access them. This is particularly important given that having the choice to disclose or not disclose is itself a privileged position and is dependent on the nature of the work, the job, the uniform requirements, or the relative autonomy and control over time and tasks that an individual has.

The findings suggest that there is a complex balance facing organizations in terms of ensuring that menopause is supported while not exposing women to further prejudice or inequality in their workplace. Similar to Grandey et al. [31] (p. 25), most women made it clear that menopause was about ‘natural and normative internal demands that women adapt to and actively cope with as part of life transitions’. It appeared that there was little correlation between the experience of their symptoms and preferences for how workplaces should treat it. In the study, three positions emerged. First, many women felt that menopause was a wholly private issue and wanted it to be kept separate from the workplace. Second were women who wanted it to be part of an explicit conversation and range of support mechanisms. Third was a group who wanted to feel supported working throughout their menopausal years, but also not feel that they must have their menopausal status ‘outed’ in order to access this support. It is the final group that is perhaps most important in terms of thinking through what successful menopause practice might consist of, particularly given that many employees could feel confident that their workplaces have given due consideration to transforming the more embedded dynamics of inequality. As one respondent poignantly put it: ‘without space for thoughts about structural issues the focus of workplace issues can be over-determined by notions of women’s biology’ (HE78).

### 4.3. Limitations

The study is not without limitations. First, the focus on workplaces meant that the sample included only women who were currently employed. While this provides valuable insights, it may overlook a hidden population of menopausal women who have already had to exit the workplace due to an inability to combine paid work with menopausal symptoms, or those trying to access paid employment while experiencing menopause. Future studies could explore what challenges or opportunities these groups of women face in terms of re-engaging with the workforce, and how menopausal-related inequities affect this. Second, by focusing on two sectors, healthcare and higher education, the results came from employees who were relatively secure and had easier access to union membership compared to the general working population. Given that there is an increasing trend towards subcontracting within many industries and sectors, future research should explore how those in precarious or low security forms of employment experience and negotiate menopausal transition. Such research might also explore the power that large organizations have to advocate for menopausal support by their subcontractors, such as making it a stipulation within the tendering process. This is particularly important given that those jobs that are likely to be subcontracted out are usually the ones with less pay, lower job security and poorer working conditions—all elements that can negatively impact health [43].

## 5. Conclusions

This study provides new insights into how multiple modes of disadvantage come together for women experiencing menopausal transition at work. It highlights that menopausal support at work can easily slip into the rhetoric of ‘managing’ menopause. Such an approach can result in consigning menopause to HR accolades, workplace awards and other short-term managerial initiatives that commoditize women’s bodies while not transforming cultures and practices over the long term. While government, policy and professional bodies are beginning to acknowledge menopause as a workplace issue, which is a positive first step, the study highlights how workplace menopausal support must challenge and change deeper structural and cultural practices to tackle embedded modes of inequality. This may involve dismantling instituted norms surrounding, for example, who undertakes what kinds of work based on the disproportionate impact it has on particular groups. Through making significant changes to how we organize work, it is likely that workplaces will not only become more menopause friendly: they will become more inclusive and sustainable for all bodies.

## Figures and Tables

**Table 1:** Overview of Key Themes.

Theme	Description	Indicative Quotations
Menopause as a slippery inequality	Inability to pinpoint the focus of discriminatory behavior (menopause, age, health or gender)	*Although at an age where menopause could be expected to be disruptive, this is not my current experience. However, I am always concerned about others’ perceptions that menopause/age may be the cause of any fatigue or frustration or other behaviors I display, when in fact I would put these down to other factors unrelated to age* (HE462) *Although I am a passionate, dedicated, vocal workers, my male colleagues could possibly think I was a menopausal middle-aged woman and disregard my concerns* (HE303)
	Menopausal transition at work experienced in combination with other identifying characteristics such as health, gender or age	*I am unsure which aspects of my health are related to menopause now* (HE352) *No, I don’t believe menopause has any effect on any aspect of my work. I often feel that some of the symptoms of menopause for me may be overtaken by the aspects of self-management that I have to do for my diabetes etc.* (HE325)
Laboring through menopause	Given roles that require high emotional labor (that can exacerbate menopausal symptoms) but are not valued institutionally	*The older nurses ended up with difficult patient allocations* (HC623) *Menopause itself is not a problem, but being over 50, female and an academic means that you are expected to take over a lot of administration. I feel like one of the old chooks who keep the place running, while younger people are given real opportunities, and much more support. The perceived value of these admin duties, of course, is close to zero. Has anyone ever got sabbatical because they put out fires with students?* (HE435)
	Lack of either flexibility to move up (development opportunities or promotion) or equitable moves to part-time hours	*It does influence perceptions of my ability and my sense of having a career trajectory. I think the older a woman is, the less likely she is to be noticed and valued in an academic workplace* (HE730) *Just because you may work only 2 days a week doesn’t mean you are to be allocated the heaviest workload on both shifts* (HC1062)
Decentering the self: Menopausal inequality through the ethos of service	Structural aspects of work create or exacerbate menopausal inequality	*Night shift is a trigger for my hot flushes and exhaustion. Takes me several days post nights to readjust to day shifts again. Often rostered to do 3 nights then 1 day off (spent sleeping after last night) and back again onto days.* (HC69) *Menopause has a huge impact on my work. The discomfort and embarrassment I feel having hot flushes is awful. I work in an unairconditioned office and this makes coping with my hot flushes even harder* (HE126)
	Service level expectations override bodily experiences of employee	*With the ever-decreasing standards and consumerization of education, I find the side effects of menopause simply make it more difficult to cope with the pressure of having to pass students and put up with their poor commitment and attitudes. However, I am not suffering as much as other women at present, therefore, I am able to have a normal working life as a lecturer. The biggest problem currently is not health but age. As an older person, I have lost any career prospects and cannot get any other type of work or positions in education* (HE416) *I don’t know when and if I will get another period but I did once when we had to wait 3 1/2 h for an ambulance. It was lucky I was wearing dark clothing. If I had been wearing light colored clothing I would have had to tell my male colleague I needed to leave. I guess there needs to be some sort of safety net for a staff member to be able to leave* (HC1014)

## Data Availability

Further information on the dataset used for this study can be found by contacting the corresponding author. Due to the sensitive nature of the data, the dataset is not publicly available.

## References

[1] Harnois C.E., Bastos J.L. (2018). Discrimination, harassment, and gendered health inequalities: Do perceptions of workplace mistreatment contribute to the gender gap in self-reported health?. J. Health Soc. Behav..

[2] Tilly J., O’Leary J., Russell G. (2013). Older Women Matter: Harnessing the Talents of Australia’s Older Female Workforce.

[3] OECD (2021). OECD Employment Outlook 2021: Navigating the COVID-19 Crisis and Recovery.

[4] Mishra G.D., Kuh D. (2012). Health symptoms during midlife in relation to menopausal transition: British prospective cohort study. Br. Med. J..

[5] Soules M.R., Sherman S., Parrott E., Rebar R., Santoro N., Utian W., Woods N. (2001). Stages of reproductive aging workshop (STRAW). J. Women’s Health Gend.-Based Med..

[6] Greene J.G. (2008). Constructing a standard climacteric scale. Maturitas.

[7] Avis N.E., Crawford S.L., Greendale G., Bromberger J.T., Everson-Rose S.A., Gold E.B., Hess R., Joffe H., Kravitz H.M., Tepper P.G. (2015). Duration of menopausal vasomotor symptoms over the menopause transition. JAMA Intern. Med..

[8] Jack G., Riach K., Bariola E., Pitts M., Schapper J., Sarrel P. (2016). Menopause in the workplace: What employers should be doing. Maturitas.

[9] Butler C. (2020). Managing the menopause through ‘abjection work’: When boobs can become embarrassingly useful, again. Work Employ. Soc..

[10] Atkinson C., Carmichael F., Duberley J. (2021). The menopause taboo at work: Examining women’s embodied experiences of menopause in the UK police service. Work Employ. Soc..

[11] Crenshaw K. (1989). Demarginalizing the intersection of race and sex: A black feminist critique of antidiscrimination doctrine, feminist theory and antiracist politics. Univ. Chic. Leg. Forum.

[12] Cho S., Crenshaw K.W., McCall L. (2013). Toward a field of intersectionality studies: Theory, applications, and praxis. Signs J. Women Cult. Soc..

[13] Crenshaw K., Fineman M.A. (1994). Mapping the margins: Intersectionality, identity politics, and violence against women of color. The Public Nature of Private Violence: Women and the Discovery of Abuse.

[14] Price K. (2011). It’s not just about abortion: Incorporating intersectionality in research about women of color and reproduction. Women’s Health Issues.

[15] Mac-Seing M., Zinszer K., Eryong B., Ajok E., Ferlatte O., Zarowsky C. (2020). The intersectional jeopardy of disability, gender and sexual and reproductive health: Experiences and recommendations of women and men with disabilities in Northern Uganda. Sex. Reprod. Health Matters.

[16] Jack G., Riach K., Hickey M., Griffiths A., Hardy C., Hunter M. (2021). Menopause in the workplace: Building evidence, changing workplaces, supporting women. Maturitas.

[17] Ryan N.E., El Ayadi A.M. (2020). A call for a gender-responsive, intersectional approach to address COVID-19. Glob. Public Health.

[18] Opara V., Sealy R., Ryan M.K. (2020). The workplace experiences of BAME professional women: Understanding experiences at the intersection. Gend. Work. Organ..

[19] Rosette A.S., de Leon R.P., Koval C.Z., Harrison D.A. (2018). Intersectionality: Connecting experiences of gender with race at work. Res. Organ. Behav..

[20] Wright T. (2013). Uncovering sexuality and gender: An intersectional examination of women’s experience in UK construction. Constr. Manag. Econ..

[21] Jammaers E., Williams J. (2021). Care for the self, overcompensation and bodily crafting: The work–life balance of disabled people. Gend. Work. Organ..

[22] Johansson M., Śliwa M. (2014). Gender, foreignness and academia: An intersectional analysis of the experiences of foreign women academics in UK business schools. Gend. Work. Organ..

[23] Anderson C. (2019). Discourses of Ageing and Gender: The Impact of Public and Private Voices on the Identity of Ageing Women.

[24] Irni S. (2009). Cranky old women? Irritation, resistance and gendering practices in work organizations. Gend. Work. Organ..

[25] (2019). Department for Educationm Skills and Employment Statistics for FTE for Full-Time and Fractional Full-Time Staff by State, Higher Education Institution, Current Duties Classification and Gender. https://www.dese.gov.au/higher-education-statistics/resources/2019-staff-fulltime-equivalence.

[26] Australian Bureau of Statistics (2020). Gender Indicators, Australia. https://www.abs.gov.au/statistics/people/people-and-communities/gender-indicators-australia/latest-release.

[27] (2021). Nursing and Midwifery Board Registrant Data 1 April to 30 June 2021. https://www.nursingmidwiferyboard.gov.au/about/statistics.aspx.

[28] OECD (2018). OECD Teaching and Learning International Survey (TALIS). https://www.oecd.org/education/talis/TALIS2018_CN_AUS.pdf.

[29] National Union of Teachers (2014). Findings of the NUT 2014 Survey on the Menopause.

[30] Bello B.G. (2016). Talking about intersectionality: Interview with Kimberlé W: Crenshaw. Sociol. Del Dirit..

[31] Grandey A.A., Gabriel A.S., King E.B. (2020). Tackling taboo topics: A review of the three M s in working women’s lives. J. Manag..

[32] Atkinson C., Beck V., Brewis J., Davies A., Duberley J. (2021). Menopause and the workplace: New directions in HRM research and HR practice. Hum. Resour. Manag. J..

[33] Tariq M., Syed J. (2017). Intersectionality at work: South Asian Muslim women’s experiences of employment and leadership in the United Kingdom. Sex Roles.

[34] Badakhsh M., Hastings-Tolsma M., Firouzkohi M., Amirshahi M., Hashemi Z.S. (2020). The lived experience of women with a high-risk pregnancy: A phenomenology investigation. Midwifery.

[35] Adolfsson A. (2010). Applying Heidegger’s interpretive phenomenology to women’s miscarriage experience. Psychol. Res. Behav. Manag..

[36] Merleau-Ponty M. (2005). Phenomenology of Perception.

[37] Van Manen M. (2016). Phenomenology of Practice: Meaning-Giving Methods in Phenomenological Research and Writing.

[38] Harlow S.D., Gass M., Hall J.E., Lobo R., Maki P., Rebar R.W., Sherman S., Sluss P.M., De Villiers T.J., Group S.C. (2012). Executive summary of the Stages of Reproductive Aging Workshop+ 10: Addressing the unfinished agenda of staging reproductive aging. J. Clin. Endocrinol. Metab..

[39] Saldaña J. (2021). The Coding Manual for Qualitative Researchers.

[40] Vardeman-Winter J., Tindall N., Jiang H. (2013). Intersectionality and publics: How exploring publics’ multiple identities questions basic public relations concepts. Public Relat. Inq..

[41] Jack G., Riach K., Bariola E. (2019). Temporality and gendered agency: Menopausal subjectivities in women’s work. Hum. Relat..

[42] Office for National Statistics (2020). Employment by Occupation. https://www.ethnicity-facts-figures.service.gov.uk/work-pay-and-benefits/employment/employment-by-occupation/latest.

[43] László K.D., Pikhart H., Kopp M.S., Bobak M., Pajak A., Malyutina S., Salavecz G., Marmot M. (2010). Job insecurity and health: A study of 16 European countries. Soc. Sci. Med..

